# Experience of acute hepatitis C and HIV co-infection in an inner city clinic in the UK

**DOI:** 10.7448/IAS.17.4.19639

**Published:** 2014-11-02

**Authors:** Christopher Ward, Vincent Lee

**Affiliations:** Manchester Centre for Sexual Health, Central Manchester University Hospitals NHS Foundation Trust, Manchester, UK

## Abstract

**Introduction:**

Acute hepatitis C infection (HCV) is increasing in the HIV-infected population, particularly among men who have sex with men (MSM). Patients co-infected with HCV and HIV progress more rapidly to liver cirrhosis and are at higher risk of hepatocellular carcinoma. We looked at our management of acute HCV to assess treatment outcome.

**Materials and Methods:**

We performed a retrospective and prospective case note review of HIV-HCV co-infected patients attending a large inner city sexual health clinic from 2006-to date. Acute HCV infections (less than six months) were identified and data was collected on demographics, transmission and treatment outcomes. Treatment regime was 48 weeks of weight-based ribavirin and pegylated interferon α2a.

**Results:**

Sixty-seven acute HCV infections were identified among 142 co-infected patients, all of whom were male and 66 (98.5%) were MSM. Median age at diagnosis was 37 (range 20–59) and 58 (86.6%) were White British. Sixty patients (89.6%) were genotype 1, 3 (4.5%) were genotype 4 and 2 (3.0%) were genotype 2/3. A further 2 (3.0%) were re-infections. A peak in new HCV diagnoses was seen in 2013 with 17 (25.4%). Route of transmission was sexual in all cases with 13 (19.4%) also injecting drugs, pointing to mixed transmission routes. Nine (69.2%) of these occurred in 2013. Nine (13.4%) patients cleared HCV themselves. Of the 58 who didn't clear HCV, 12 (20.7%) were lost to follow up/transferred care, 4 (6.9%) declined treatment awaiting newer agents, and 10 (17.2%) are waiting to start. A total of 32 patients started treatment. Six (18.8%) patients are currently on treatment and three (9.4%) await a final sustained virological response (SVR) test. Six out of twenty-four (25.0%) stopped treatment due to lack of response and 1 stopped due to side effects. Fifteen (62.5%) achieved SVR and 2 (8.3%) failed to achieve SVR. Eight out of ten (80.0%) patients who had an early virological response (EVR) achieved SVR.

**Conclusions:**

Our data shows good treatment outcomes for acute HCV infection in HIV patients with an SVR rate of 62.5%. We've seen a steady increase in acute HCV infection, particularly in MSM injecting party drugs. Changing risk behaviours, particularly a rise in chem sex parties and club drug use, along with more anonymous partners and disclosure issues create difficulties in managing the HCV epidemic. More education is needed to raise awareness of HCV transmission and disclosure in our MSM population.

